# Combined Multivariate Statistical Techniques, Water Pollution Index (WPI) and Daniel Trend Test Methods to Evaluate Temporal and Spatial Variations and Trends of Water Quality at Shanchong River in the Northwest Basin of Lake Fuxian, China

**DOI:** 10.1371/journal.pone.0118590

**Published:** 2015-04-02

**Authors:** Quan Wang, Xianhua Wu, Bin Zhao, Jie Qin, Tingchun Peng

**Affiliations:** Yuxi Research Center for Eco-environmental Sciences on Plateau Lakes, Yuxi Normal University, Yuxi, Yunnan Province, P.R. China; University of Waikato (National Institute of Water and Atmospheric Research), NEW ZEALAND

## Abstract

Understanding spatial and temporal variations in river water quality and quantitatively evaluating the trend of changes are important in order to study and efficiently manage water resources. In this study, an analysis of Water Pollution Index (WPI), Daniel Trend Test, Cluster Analysis and Discriminant Analysis are applied as an integrated approach to quantitatively explore the spatial and temporal variations and the latent sources of water pollution in the Shanchong River basin, Northwest Basin of Lake Fuxian, China. We group all field surveys into 2 clusters (dry season and rainy season). Moreover, 14 sampling sites have been grouped into 3 clusters for the rainy season (highly polluted, moderately polluted and less polluted sites) and 2 clusters for the dry season (highly polluted and less polluted sites) based on their similarities and the level of pollution during the two seasons. The results show that the main trend of pollution was aggravated during the transition from the dry to the rainy season. The Water Pollution Index of Total Nitrogen is the highest of all pollution parameters, whereas the Chemical Oxygen Demand (Chromium) is the lowest. Our results also show that the main sources of pollution are farming activities alongside the Shanchong River, soil erosion and fish culture at Shanchong River reservoir area and domestic sewage from scattered rural residential area. Our results suggest that strategies to prevent water pollutionat the Shanchong River basin need to focus on non-point pollution control by employing appropriate fertilizer formulas in farming, and take the measures of soil and water conservation at Shanchong reservoir area, and purifying sewage from scattered villages.

## Introduction

The demand of freshwater and the deterioration of water quality have both rapidly increased in China[1–5]. As the largest deep fresh lake and the important freshwater resources in China, Lake Fuxian is very important in Yunnan province, even in China. The water quality of this lake is better than the drinking water sources that corresponds to Class II of China National Water Quality Standard (**Table 1**)[6,7]. The recharge water of Lake Fuxian mainly comes from the inflow rivers. However, most of the inflow rivers are polluted seriously. The water quality of these rivers is worse than landscape water that correspond to Class V of the China National Water Quality Standard and the main parameters which exceed the standard are Total Nitrogen (TN) and Total Phosphorus (TP),[8,9] which are the main factors of eutrophication[10–13]. Improving the water quality of the inflow rivers is therefore essential for protecting the water quality of Lake Fuxian.

**Table 1:** China National Water Quality Standard (CNWQS) and basic statistical information and monitoring method of the water quality parameters in the Shanchong river basin.

Parameters	Mean±SD	Environmental guides					Analytical methods
		First level	Second level	Third level	Fourth level	Fifth level	
**TP(mg/L)**	0.56±0.89	0.02	0.10	0.20	0.30	0.40	Molybdenum antimony anti Spectrophotometry
**TN(mg/L)**	10.05±10.75	0.20	0.50	1.00	1.50	2.00	Potassium persulfate UV Spectrophotometry
**NH**_**3**_ **-N(mg/L)**	2.56±6.3	0.15	0.50	1.00	1.50	2.00	Nessler's reagent spectrophotometric
**COD**_**cr**_ **(mg/L)**	18.52±10.49	15.00	15.00	20.00	30.00	40.00	Dichromate titration

About 1.4 billion-RMB (US$225-million) have been spent on protecting Lake Fuxian from pollution during the past 17 years[14], but the water quality of inflow rivers was still worse than class Ⅴ. The local government has starting a three-years (2014–2016) and 10.5-billion-RMB (US$1.686-billion) program for environmental conservation of Lake Fuxian. The focus is on the comprehensive improvement of water environment of inflow rivers, the construction wetland and the non-point source pollution control.It will help to take the proper pollution control action and avoid funds waste if we have the information about main pollution source and the spatial-temporal regulation of contamination occurred. [1,3,15–19]. Few studies have investigated the pollution of the inflow rivers of the Lake Fuxian.[8,9,20] Furthermore, the spatial-temporal variations and trends of river water quality have not been fully explored in the literature for the inflow rivers of the Lake Fuxian. [20]

A Daniel Trend Test provides a quantitative evaluation of the changing trend. It makes the change easy to understand[20–26]. One of the main contributions of this paper is to investigate both temporal and linear spatial variation of water pollution at the Shanchong river basin by using the Daniel Trend Test method. The Water Pollution Index (WPI) is recommended by Ministry of Environmental Protection of the People's Republic of China to evaluate the water pollution[27]. We use the WPI to compare the pollution level between different pollution parameters and reveal the main pollution parameters in this study [20,25,27]. Cluster Analysis and Discriminant Analysis are multivariate techniques which are normally used to group objects into classes consisting of similar features. Meanwhile, these techniques have been widely used in river water quality assessment[15,28–36].

In this study, we apply different statistical techniques to a data matrix obtained from March to October in 2013 to extract information about the spatio-temporal variations and trends in water quality parameters. The information is quantitatively evaluated by Daniel trend test. The main pollution parameter is found through WPI method. The pollution source is analyzed, and the spatio-temporal grouping which produced by Cluster Analysis and Discriminant Analysis is used to interpret the distribution of pollution in spatioal and temporal. The result could be helpful for effective water quality management at the Shanchong river basin.

## Materials and Methods

### Ethics statement

No specific permits were required for the described field studies and our field studies did not involve endangered or protected species.

### Study area

**Fig 1:**
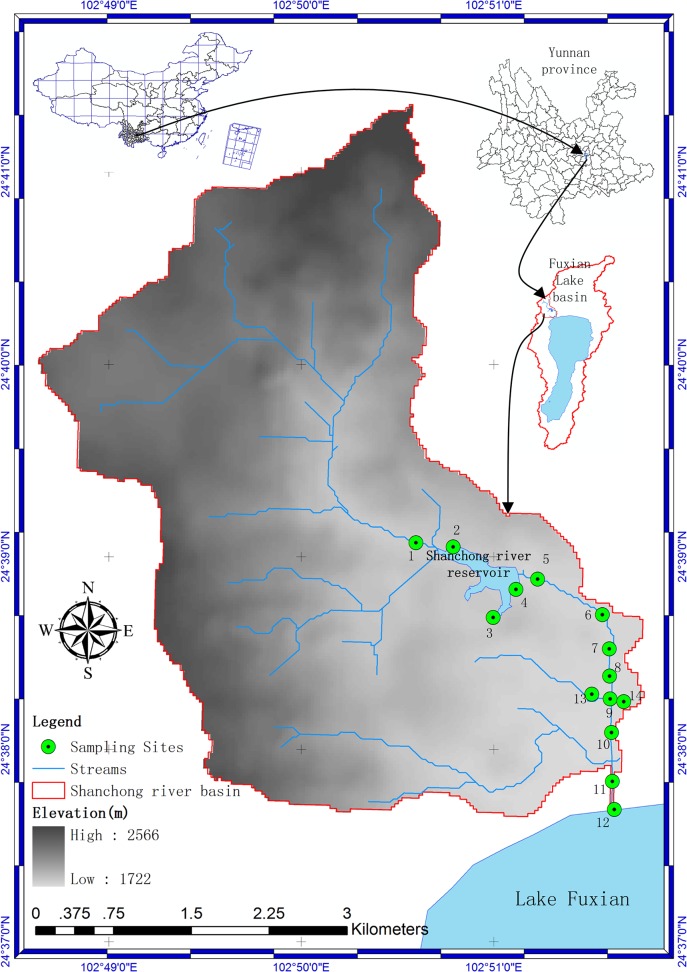
The Geographical Location of The Sampling Sites in the Shanchong River Basin.

The Shanchong River (24°37’—24°41’ N, 102°48’–102°50’ E; 1703–2606 m Latitude, Longitude, Elevation), is situated northwest of Lake Fuxian with a drainage area of approximately 19.42 km^2^ and the stream is 29.05 km long (**Fig. 1**). The dry and rainy season is trenchant at Shanchong River Basin, and the rainy season generally from May to October. The cumulative precipitation from November, 2012 to April, 2013 were 41.5mm and that were 755.97mm from May, 2013 to October, 2013 at our study area (According to the the dataset of China daily grid precipitation which provide by China Meteorological Data Sharing Service System, http://cdc.cma.gov.cn). The dominant land cover category is cultivated land. The soil type of Shanchong river basin is Cumulic Anthrosols and the average of soil pH, soil's available phosphorus content, soil's available nitrogen content and soil organic matter were 7.45, 14.77 mg/kg, 98.37 mg/kg, and 29.62 g/kg, respectively [37,38]. The Shanchong river reservoir is located at the middle of Shanchong river basin with an area of approximately 0.154 km^2^. Furthermore, it is the main source of agricultural irrigation water for the lower reaches.

### Data sources

7 field surveys (March, April, May, Jun, July, September and October, 2013) were implemented at 14 sampling sites, of which the first survey in March only include 12 sites due to the lack of water in the river at sampling sites 5# and 6#. For the same reason there were 9, 13, 11, 10, 13 and 13 sampling sites in the April, May, Jun, July, September and October survey, respectively. Criteria of sampling sites setting, there is eight sampling sites (5#–12#) staying in the Shanchong river from upstream to downstream and there are about 500 m between every two sites, while appropriate adjusting the position of some sites by considering the sampling convenience. One (1#) sampling site stays in upstream of the Shanchong river reservoir, three sampling sites (2#–4#) stay around the Shanchong river reservoir and another two (13# and 14#) stay in the tributary of the Shanchong river. In the upstream of 13# site there are a village and farmland and which is very close to the village, about 20 m. Moreover, in the upstream of 14# site there is just farmland. The water samples were collected at a depth of 10 cm, placed into plastic bottles (2.5 L), transported to the laboratory and stored at 0–4°C for subsequent chemical analysis. The chemical measurements were performed in the laboratory within 24 hours after the collection of the water samples. There are 4 water quality parameters –Total Nitrogen (TN), Total Phosphorus (TP), Chemical Oxygen Demand (Chromium)(COD_cr_) and Ammonia Nitrogen (NH_3_-N). They have been monitored by using standard methods (**Table 1**)[39]. The China National Water Quality Standard (CNWQS)[7] and thebasic statistical information and monitoring method of the water quality parameters in the Shanchong river basin are summarized in Table 1.

### Data analytical methods

**Water Pollution Index.** The calculation of WPI is based on the water quality standard (Class I-V), for example: the water quality standard class V of TP is 0.4mg/L, and class V of TN is 2.0mg/L, if the concentration of TP is 0.4 mg/L, the WPI of TP is 100, and if the concentration of TN is 2 mg/L, the WPI of TN is 100 as well. So, the WPI can use to compare the pollution level between different pollution parameters and reveal the main pollution parameters [20,25,27].

In this study, all raw concentration data of pollution parameters from the monitoring are calculated and transformed to the Water Pollution Index (WPI) that best explains the main pollution factor and allows for easy comparison. This is done according to the Technical Guideline for Surface Water Environmental Quality Assessment(HJ422-2008)[27], and defined as:

$$WPI(i)=WPI_{l}(i)+\frac{WPI_{h}(i)-WPI_{l}(i)}{C_{h}(i)-C_{l}(i)}\times (C(i)-C_{l}(i))\;C_{l}(i)<C(i)\leq C_{h}(i)$$ (1)

where *C (i)* is the monitoring concentration of water quality parameter i;

*C*_*l*_ *(i)* is the concentration of prescribed minimum in the category to which water quality parameter i belongs;

*C*_*h*_ *(i)* is the concentration of prescribed maximum in the category to which water quality parameter i belongs;

*WPI*_*l*_ *(i)* is the index of the prescribed minimum in the category to which water quality parameter i belongs;

*WPI*_*h*_ *(i)* is the index of the prescribed maximum in the category to which water quality parameter i belongs; and

*WPI (i)* is the index of water quality parameter i.

When the monitoring concentration of water quality parameter is beyond Class Ⅴ(GB3838-2002)(7), the WPI computer equation is

$$WPI(i)=100+\frac{C(i)-C_{5}(i)}{C_{5}(i)}\cdot 40$$ (2)

where C_5_ (*i*) is the upper limit of Class V (GB3838-2002)[7] of water quality parameter i.

The WPI is equal to 20 when the monitoring concentration of water quality parameter belong to Class I (GB3838-2002)[7].

**Daniel Trend Test.** The Daniel Trend Test method is usually used in analysing temporal variation trends[20–26]. In fact, linear spatial series (such as upstream to downstream of a river) are similar to temporal series in terms of that from the past to the present time is a linear sequence as well. However, just one study has used Daniel Trend Test to explore spatial variation trend[20].

According to the Technical Guideline for Surface Water Environmental Quality Assessment (HJ422-2008)[27], the quantitative evaluation of the variation tendency of temporal and spatial of water quality is done by means of Daniel Trend Test. This method belongs to the class of non-parametric tests and adopts Spearman rank correlation coefficient to inspect the significance of changes in trend. We use the raw concentration data at Daniel Trend Test calculation. More specifically,

$$\begin{array}{l} R_{s}=1-[6\sum_{i=1}^{N}D^{2}i]/(N^{3}-N)\\ D_{i}=X_{i}-Y_{i} \end{array}$$

where *R*_*s*_ is the rank correlation coefficient, *D*_*i*_ is the difference between *X*_*i*_ and *Y*_*i*_. *X*_*i*_ is the order number (from small to big) of the raw concentration value of water sample from 1 to N., *Y*_*i*_ is the order number arranged by time sequence number or the spatial arrangement of the serial number, and *N* is the number of sampling sites or the number of periods.

The trend is aggravated or increased if the *R*_*s*_ is greater than zero, and *R*_*s*_ is less than zero if the trend is the opposite. Furthermore, the trend is significant if the *R*_*s*_ is beyond the W_p_ that is the critical value of *R*_*s*_ (**Table 2 and Table 3**).

**Table 2:** Temporal variation of each sampling sites calculated by Daniel trend test method in the Shanchong river basin.

Sampling Sites	N	Wp^a^	*R*_*s*_ (TP)	*R*_*s*_ (TN)	*R*_*s*_ (NH_3_-N)	*R*_*s*_ (COD_cr_)
**1#**	7	0.714	0.286	**0.750**	0.036	0.179
**2#**	7	0.714	0.107	0.357	**0.786**	−0.357
**3#**	7	0.714	−0.036	**0.750**	0.679	0.571
**4#**	7	0.714	0.250	−0.143	0.643	0.000
**8#**	5	0.9	0.600	−0.300	−0.300	0.200
**9#**	7	0.714	0.500	0.536	0.214	0.143
**10#**	7	0.714	0.214	0.214	0.393	−0.107
**11#**	7	0.714	0.036	0.179	0.286	0.607
**12#**	7	0.714	0.643	0.679	0.000	0.143
**13#**	7	0.714	0.500	**0.786**	0.357	0.643

^a^ critical value of *R*_*s*_ (significant level of unilateral test 0.05)

The minimum data requirements of Daniel trend test is 5, but The N of 5#, 6#, 7# and 14# are 3, 2, 4 and 4, respectively. So they are not suitable to analysis in the Daniel trend test method.

**Table 3:** Spatial variation of each field survey calculated by Daniel trend test method in the Shanchong river basin.

Date	Parameter	N	W_p_ ^a^	*R*_*s*_
Mar	TP	10	0.564	−0.091
	TN	10	0.564	0.539
	NH_3_-N	10	0.564	0.273
	COD_cr_	10	0.564	−0.32
Apr	TP	8	0.643	−0.333
	TN	8	0.643	0.857
	NH_3_-N	8	0.643	0.952
	COD_cr_	8	0.643	0.714
May	TP	11	0.506–0.564^b^	0.273
	TN	11	0.506–0.564^b^	0.936
	NH_3_-N	11	0.506–0.564^b^	0.664
	COD_cr_	11	0.506–0.564^b^	0.200
Jun	TP	10	0.564	0.030
	TN	10	0.564	0.479
	NH_3_-N	10	0.564	0.794
	COD_cr_	10	0.564	−0.042
Jul	TP	8	0.643	−0.667
	TN	8	0.643	0.643
	NH_3_-N	8	0.643	0.333
	COD_cr_	8	0.643	0.571
Sep	TP	11	0.506–0.564^b^	0.164
	TN	11	0.506–0.564^b^	0.818
	NH_3_-N	11	0.506–0.564^b^	0.709
	COD_cr_	11	0.506–0.564^b^	−0.036
Oct	TP	12	0.506	0.294
	TN	12	0.506	0.573
	NH_3_-N	12	0.506	0.825
	COD_cr_	12	0.506	−0.154

^a^ critical value of *R*_*s*_ (significant level of unilateral test 0.05)

^b^ The W_p_ is 0.564 when N is 10, moreover, the W_p_ is 0.506 when N is 12.

**Multivariate Statistics Method**. Hierarchical Cluster Analysis is a widely used cluster analysis method[30–33], and we employed this approach by means of the Ward ’s method using Squared Euclidean distance as a measure of similarity in the present study. Discriminant Analysis is performed on each data matrix using stepwise method in constructing discriminant functions to evaluate both the spatial and temporal variations in river water quality of the basin. Spatial-temporal clustering analysis was be done in our study.

Usually, the raw data used for Cluster Analysis should be transformed in order to eliminate the influence of variable dimension. But there is no need to transform the data if the WPI data are used in the analysis instead of the raw concentration data, because WPI has no difference in dimension and allow for comparison among of different water quality parameters.The data used to Temporal Hierarchical Cluster Analysis are mean value of WPI of all water samples at each field survey, and the data used to Spatial Hierarchical Cluster Analysis are mean value of WPI of each cluster generated by Temporal Hierarchical Cluster Analysis.

All the statistical analyses are performed using SPSS 18.0 for windows and Excel 2010 for windows. Latitude and longitude were measured using a hand-held GPS (MAGELLAN eXplorist 500). The Shanchong river basin boundaries were delineated on a 30-m spatial resolution digital elevation model (DEM); the stream network was represented using the Soil and Water Assessment Tool (SWAT) model at the ArcGIS 9.3 Desktop GIS software.

## Results and Discussion

### Temporal variations trend

**Fig 2:**
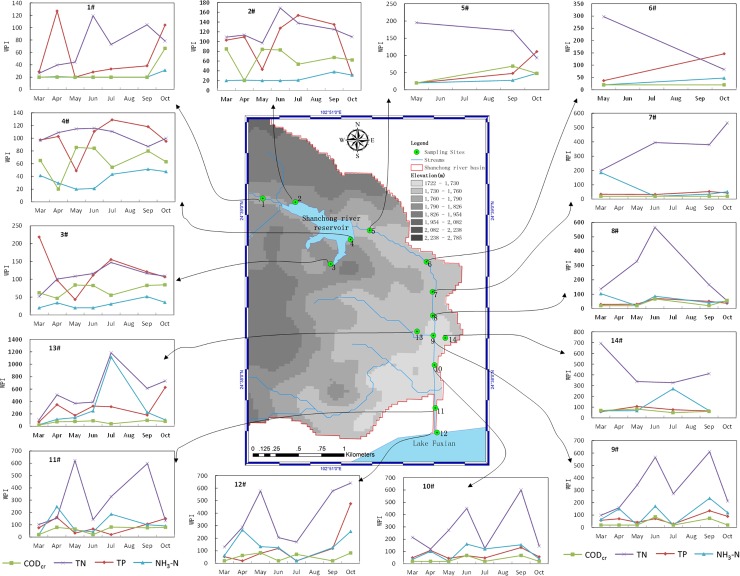
Temporal Variation of WPI in each Sampling Sites In The Shanchong River Basin.

**Fig. 2** shows the temporal variation of WPI in each sampling site. A key finding is that the WPI of TN is almost the highest of all sites in all field surveys. It indicates that nitrogen pollution is more serious than phosphorus pollution at Shanchong river basin. The reason may be the soil phosphorus content, the excess fertilization and proportion of nitrogen fertilizer and phosphorus fertilizer used at our research area. The research from B.Lars, et al showed that the leaching of nitrate increased sharply when the use of nitrogen fertilizer exceed 100 kg/ha[40]. There are about 190 kg/ha nitrogen fertilizer applied at Yuxi district where Shanchong river basin locates [41]. Therefore, leaching of nitrate may increase substantially at our research area. Another long term research showed that leaching of phosphorus linear increased when soil's available phosphorus content exceeds 60 mg/kg soil[42]. Therefore, the leaching of phosphorus was moderate at our research area because the soil's available phosphorus content are between 1.6–40.7mg/kg soil in this area[37]. According to the Yunnan statistical yearbook 2013[41], the proportion of nitrogen fertilizer and phosphorus fertilizer is 5:1 at Yuxi district[37], this may be another reason that nitrogen pollution excess the phosphorus at our study area.

The Daniel Trend Test shows that the temporal variation of all sampling sites are clear. From **Table 2**, which shows that the WPI of most monitoring parameters were increased (*R*_*s*_>0) from March to October. However, just a few are decreased (*R*_*s*_<0), which are COD_cr_(2# and 10#), NH_3_-N(8#),TN(4# and 8#) and TP(3#). It shows that the main trend of pollution is aggravated from the dry to the rainy season. The reason is rainfall in the rainy season accounts for 94.8% of rainfall throughout the year (According to the the dataset of China daily grid precipitation which provide by China Meteorological Data Sharing Service System, http://cdc.cma.gov.cn), and rainfall erosion pollutants containing nitrogen and phosphorus from farmland soil to river.

**Fig 3:**
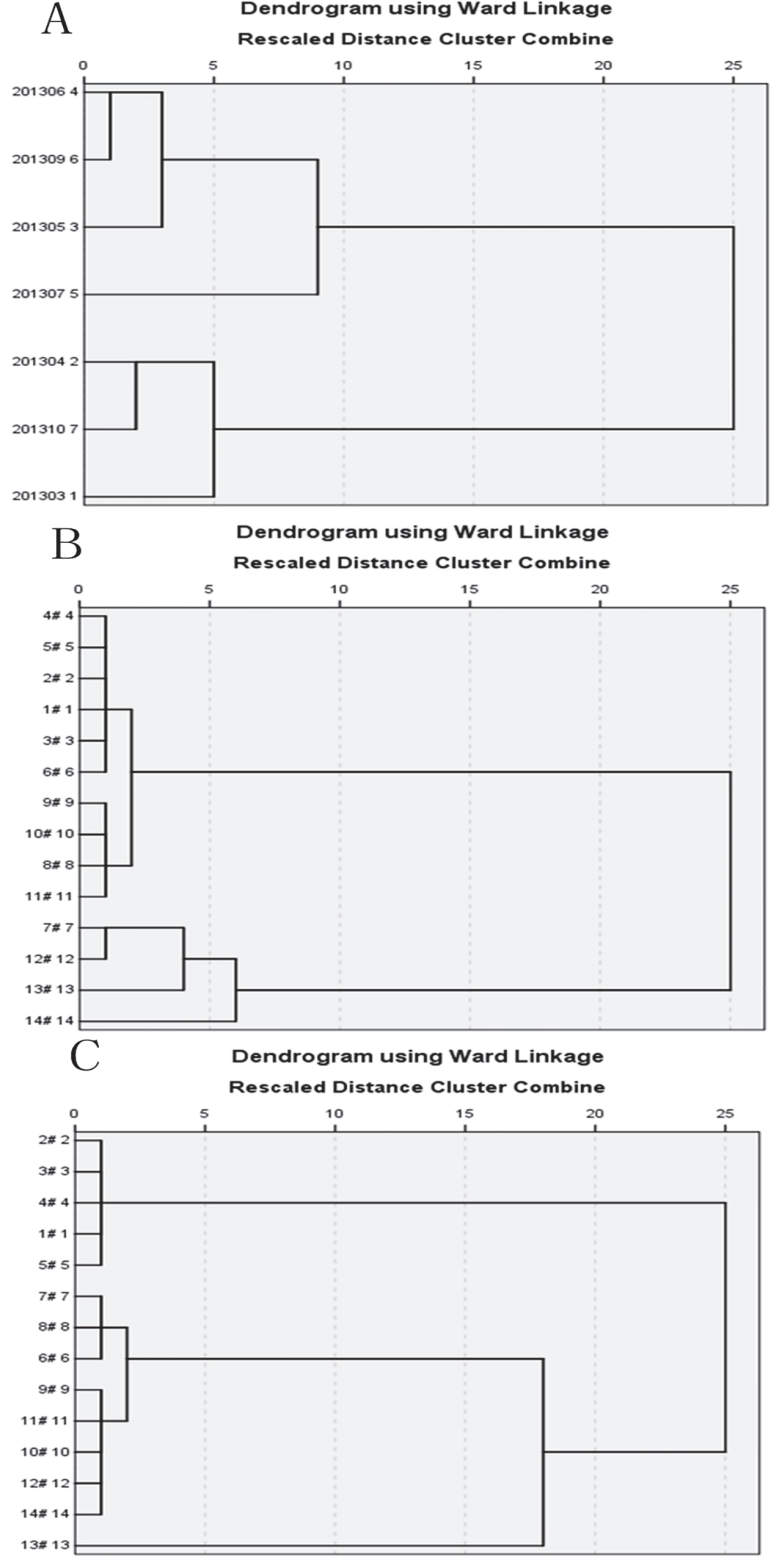
The result of Cluster Analysis In The Shanchong River Basin. (A: Hierarchical Cluster Analysis used in temporal variations.; B: Hierarchical Cluster Analysis in the Dry Season.; C: Hierarchical Cluster Analysis in the Rainy Season)

Hierarchical Cluster Analysis used in temporal variations yields a dendrogram (**Fig. 3A**), 7 months were grouped into two clusters. Cluster 1 (the first period) includes May, June, July and September, which corresponds to the rainy season. Additionally, cluster 2 (the second period) includes March, April and October, which corresponds to the dry season, with the exception of October. Specifically, most of the pollutants have been washed away by the earlier runoff and reduce contamination in October, although it belongs to the rainy season. Therefore, 7 months are divided into two different clusters (rainy season and dry season). This temporal pattern of water quality actually makes more sense because of the obvious discrimination between the rainy season and dry season, and the water quality is mainly affected by the non-point source pollution in the present study area.

Discriminant Analysis is used to further evaluate Temporal variations in water quality. Discriminant Analysis of temporal variation is performed after the whole data set is divided into two seasonal groups (rainy season and dry season). classification matrices (CMs) and Discriminant functions (DFs) obtained from the stepwise method are shown in **Table 4**. The stepwise mode DFs using 2 discriminant variables, and yield the corresponding CMs assigning 100% of the cases correctly. Thus, the Discriminant Analysis results of temporal variation suggest that TN and TP are the most significant parameters to discriminate between two periods, which means that these two parameters account for most of the expected temporal variations in the river water quality (**Table 4**).

**Table 4:** Classification functions for discriminant analysis of spatial and temporal variation in water quality of the Shanchong river basin.

Parameters	Temporal		Spatial				
	dry season^a^	rainy season^b^	Dry season		Rain season		
			less polluted sites ^c^	highly polluted sites^d^	less polluted sites ^e^	moderately polluted sites^f^	highly polluted sites^g^
TN	0.179	0.401	0.022	0.084	0.074	0.22	0.279
NH_3_-N	-	-	0.048	0.12	−0.014	−0.041	0.218
TP	−0.011	−0.193	-	-	-	-	-
Constant	−17.101	−53.202	−3.268	−27.033	−5.557	−40.932	−137.297

Fisher's linear discriminant functions

^a^ dry season includes March, April and October.

^b^ rainy season includes May, June, July and September.

^c^ less polluted sites(1#–6# and 8#–11#).

^d^ highly polluted sites(12#–14# and 7#).

^e^ less polluted sites(1#–5#).

^f^ moderately polluted sites(6#–12# and 14#).

^g^ highly polluted sites(13#).

**Fig 4:**
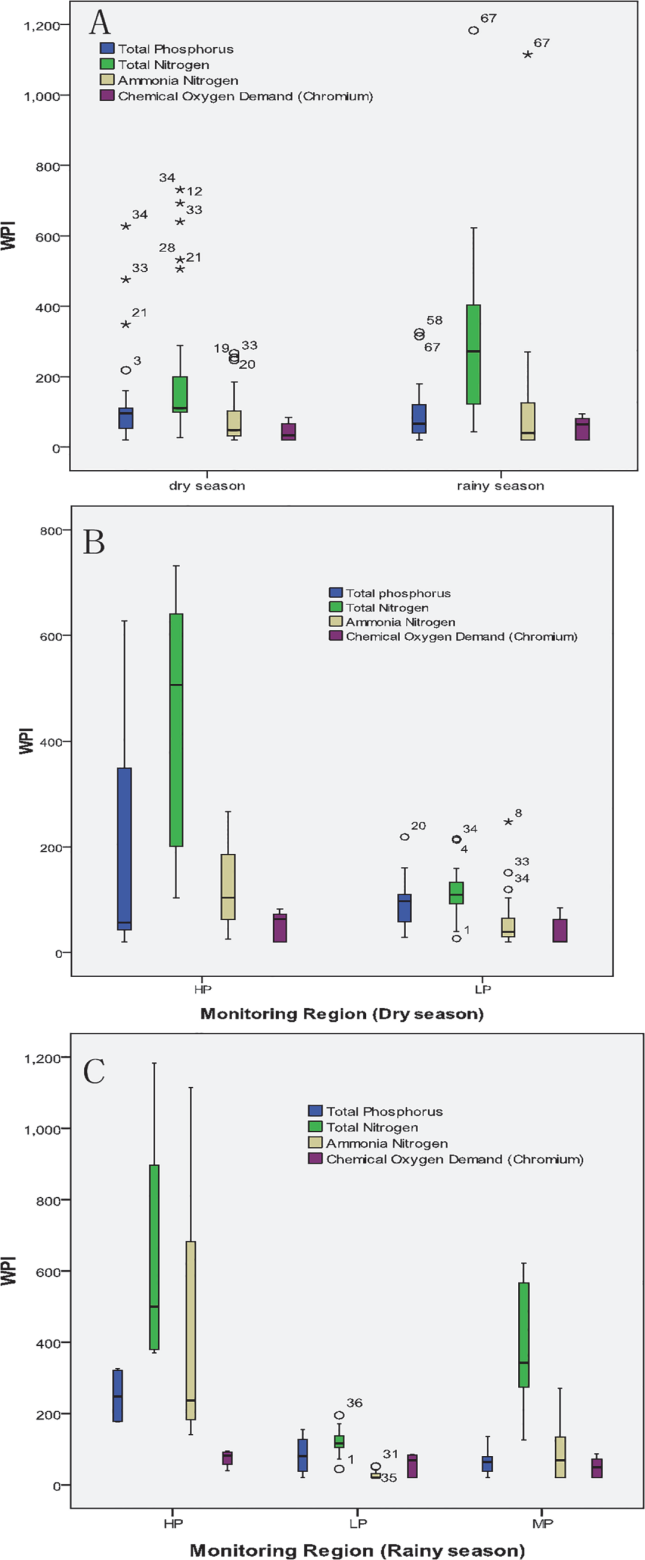
Box and Whisker Plots of Temporal and Spatial Variation In The Shanchong River Basin.

Box and whisker plots of the all parameters showing temporal variation are given in **Fig. 4A**. The WPI of TN was higher in the rainy season as compared to the dry season obviously, whereas, TP, NH_3_-N and COD_cr_ have little difference in the present study area.

### Spatial variations trend

**Fig 5:**
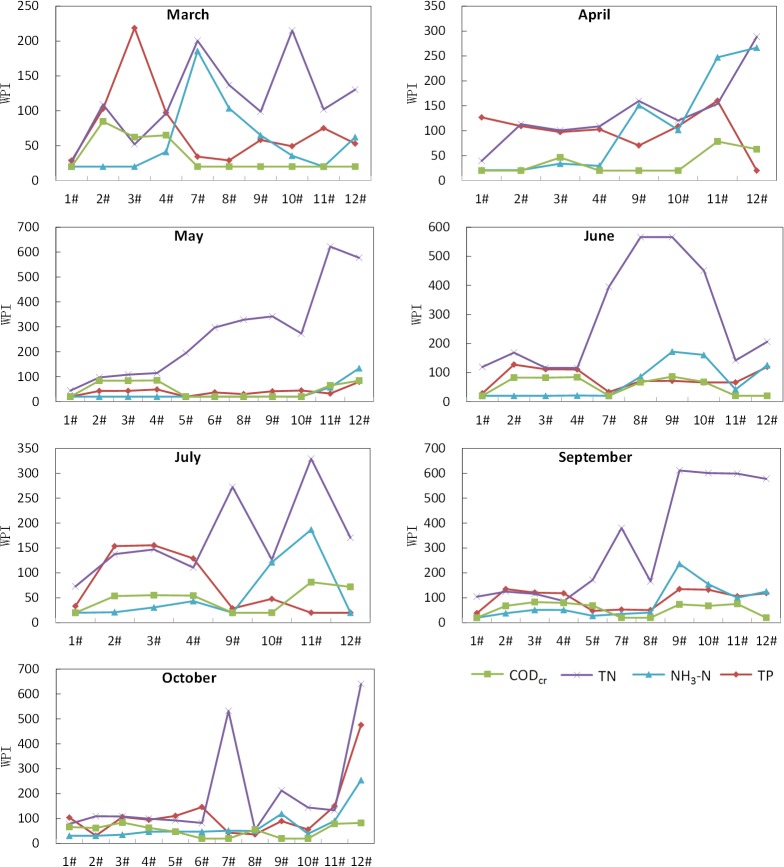
Spatial Variation of WPI in each Field survey In The Shanchong River Basin.

The spatial variation of WPI in each field survey from upstream to downstream along the river are shown in **Fig. 5**. The data used to analyse spatial variation of WPI come from 1#-12# sampling sites which were all in the Shanchong river. A behavior similar to that described for temporal variation was observed again, as the WPI of TN was almost the highest after May, whereas the WPI of CODcr was always the lowest compared to other pollution parameters. Indeed, it is not easy to note a clear trend of either increase or decrease in the values of WPI, especially at March and April. The Daniel Trend Test could provide a quantitative evaluation of the change in trend. According to **Table 3**, the spatial variation of each field survey is clear, in the sence that the result shows that the WPI of most monitor parameters are increased (*R*_*s*_>0) from upstream to downstream. Specifically, just a few are decreased (*R*_*s*_<0) which were TP (March, April and July) and CODcr (March, June, September and October). It indicates that the main spatial trend of pollution is aggravated from upstream to downstream. The reason may be the rainfall erosion pollutants from farmland soil to river, meanwhile, leaching of containment matters to ground water, finally may effect the downstream of river, make pollution aggravated from upstream to downstream of the river.

Hierarchical Cluster Analysis is used in spatial variations to detect the similarity of the groups between the sampling sites. We use Spatial Hierarchical Cluster Analysis in the rainy season and dry season, respectively. This analysis yields a Dendrogram (**Fig. 3B** and **Fig. 3C**), grouping all 14 sampling sites of the basin into three statistically significant clusters in the rainy season (**Fig. 3C**) and two statistically significant clusters in the dry season (**Fig. 3B**).

In the dry season, cluster 1 (1#–6# and 8#–11#) corresponds to relatively less polluted sites, of which five stations (1#–5#) are situated at the upstream sites and 6#, 8#-11# are situated at the downstream site of the river. In cluster 1, the group suggests the assimilative capacity and self purification of the river. Additionally, Cluster 2 (12#–14# and 7#) corresponds to relatively highly polluted sites which are situated at middle of the river and receive pollution mostly from domestic wastewater.

In the rainy season, cluster 1 (1#–5#) corresponds to relatively less polluted sites and is situated at the upstream sites where around Shanchong river reservoir with scarcity of the people and farmland. Additionally, cluster 2 (6#–12# and 14#) corresponds to relatively moderately polluted sites and these stations receive pollution from the sources of agricultural activities, which is the typically non-point source. Lastly, cluster 3 (only 13#) corresponds to relatively highly polluted sites and this station receive pollutes from domestic wastewater and non-point sources. Furthermore, we find the sites 10, 11 and 12 which stays close to outlet of Shanchong river have in general higher amounts of TN during all seasons. The reason may be the excess nitrogen fertilization and the non-point source pollution which have been discussed above.

Spatial variations in water quality are further evaluated through Discriminant Analysis. Spatial Discriminant Analysis is performed after dividing the whole data set into two major classes of less polluted and highly polluted sites as obtained through Cluster Analysis of the dry season and three major classes of less polluted, moderately polluted and highly polluted sites as obtained through Cluster Analysis of the rainy season. The stepwise method provides Discriminant functions (DFs) and classification matrices (CMs), which are shown in **Table 4**. The stepwise mode DFs using 2 discriminant variables (TN and NH_3_-N) yield the corresponding CMs assigning 100% (dry season) and 92.9% (rainy season) of the cases correctly. Furthermore, the spatial Discriminant Analysis results suggest that TN, NH_3_-N are the most significant parameters to discriminate different spatial groups both in dry and rainy season, which means that these two parameters account for most of the expected spatial variations in the river water quality (**Table 4**).

Box and whisker plots of the all parameters showing spatial variations are given in Fig. 4B and Fig. 4C. The monitoring region has been class to highly polluted and less polluted sites in the dry season (**Fig. 4B**). Moreover, the WPI of TN, NH_3_-N and TP are obviously higher in highly polluted sites as compared to less polluted sites in the dry season. Furthermore, the WPI of COD_cr_ which has the lowest pollution parameters compare to other parameters and it has little difference between the less polluted and highly polluted sites.

Additionally, all sampling sites have been class to highly polluted, moderately polluted and less polluted sites in the rainy season. A decrease in WPI of TN and NH_3_-N (**Fig. 4C**) from highly polluted to less polluted sites is observed in the rainy season. The WPI of TP at less polluted sites is between the moderately polluted sites and highly polluted sites. The WPI of COD_cr_ is slightly different among highly polluted, moderately polluted and less polluted sites. The possible reason is the fish culture inside the Shanchong river reservoir and furthermore, the fragile ecosystem around the Shanchong river reservoir basin which has a lot of eucalyptus and almost without shrubs and herbs around it and made bare in surface. Furthermore, the slope of the hill which around the Shanchong river reservoir is greater than 45°. Finally, these factors aggravate soil erosion. It may be the cause of phosphorus pollution at less polluted sites seriously than moderately polluted sites.

## Conclusions

The primary results of this study can be summarized as follows:

(1)The Hierarchical Cluster Analysis that is used in temporal variations generates two clusters by their hydrological characteristics (rainy season and dry season). Moreover, the TN and TP are the most significant parameters to discriminate between the two periods.

(2)The Hierarchical Cluster Analysis that is used in spatial variations, groups all 14 sampling sites of the basin into three statistically significant clusters (highly polluted, moderately polluted and less polluted sites) in the rainy season and two statistically significant clusters (highly polluted and less polluted sites) in the dry season. Furthermore, the TN and NH_3_-N are the most significant parameters to discriminate different spatial groups both in the dry and rainy season.

(3)The main trend in pollution is aggravated from the dry to the rainy season and the same spatial trend from upstream to downstream.

(4) The WPI of TN is the highest of all pollution parameters, whereas the CODcr is the lowest. The WPI of TN is obviously higher in the rainy season as compared to the dry season, whereas, the water quality parameters of TP, NH_3_-N and CODcr have little difference between the rainy season and dry season. The possible reason is the excess nitrogen fertilization and non-point source pollution.

(5) The WPI of TN, NH_3_-N and TP are obviously higher in highly polluted sites (situated at middle of river and received pollution mostly from domestic wastewater) as compared to less polluted sites (situated at the upstream around Shanchong river reservoir and downstream nearly steam outlet) in the dry season. Moreover, a decrease in WPI of TN and NH_3_-N from highly polluted sites (13# that situated at middle of river) to less polluted sites (situated at the upstream around Shanchong river reservoir) is observed at the rainy season. The WPI of TP at less polluted sites is between the moderately polluted sites (situated at downstream) and the highly polluted sites. The possible reason was the fish culture and the aggravating soil erosion at less polluted area.

(6)The main pollution factors are non-point source from farming activities along the Shanchong river, soil erosion, and fish culture at Shanchong river reservoir area, and the domestic sewage from rural residential area.

(7)The Daniel Trend Test method is usually used in the analysis of temporal variation trends. Because the linear spatial series (such as upstream to downstream of river) has the same characteristics as the temporal series, in this study we use the Daniel Trend Test to evaluate both temporal and spatial variation trends of water quality of the Shanchong river. It implicates that this method is the proper tool for evaluating the linear spatial series data.

Our results suggest that adopting water pollution prevention strategies of the Shanchong river basin should be in three directions. First, to employ appropriate fertilizer formulas in farming to cut down non-point source pollution. Second, to take the measure of soil and water conservation at Shanchong reservoir area to prevent the deterioration of water quality of the reservoir. Third, to construct scattered sewage treatment system at rural residential area to purification of sewage.

## References

[1] Chen J, Lu J. Effects of Land Use, Topography and Socio-Economic Factors on River Water Quality in a Mountainous Watershed with Intensive Agricultural Production in East China. PLoS ONE. 2014;9:e102714 10.1371/journal.pone.0102714 25090375 PMC4121078

[2] Li S, Gu S, Liu W, Han H, Zhang Q. Water quality in relation to land use and land cover in the upper Han River Basin, China. CATENA. 2008;75:216–222. 10.1016/j.catena.2008.06.005

[3] Zhang Y, Guo F, Meng W, Wang X-Q. Water quality assessment and source identification of Daliao river basin using multivariate statistical methods. Environ Monit Assess. 2009;152:105–121. 10.1007/s10661-008-0300-z 18523854

[4] Zhou N, Westrich B, Jiang S, Wang Y. A coupling simulation based on a hydrodynamics and water quality model of the Pearl River Delta, China. J Hydrol. 2011;396:267–276. 10.1016/j.jhydrol.2010.11.019

[5] Tao T, Xin K. Public health: A sustainable plan for China’s drinking water. Nature. 2014;511:527–528. 10.1038/511527a 25079539

[6] Lei Zhao XZ. Three-dimensional hydrodynamic and water quality model for TMDL development of Lake Fuxian, China. J Environ Sci China. 2012;24:1355–1363. 10.1016/S1001-0742(11)60967-4 23513675

[7] Ministry of Environmental Protection of the People’s Republic of China. China National Water Quality Standard(GB3838-2002). Beijing: China: Environmental Science Press.; 2003.

[8] Bin Wu, Jie Qin, Hong Guo, Xianhua Wu. Impacts of Main Inflow Rivers on North Bank of Lake Fuxian on Water Quality of the Lake. J Yuxi Norm Univ. 2010;26:39–42.

[9] Lin Wang, Xianhua Wu, Hong Guo, Xianxue Wu. Analysis of Water in tributaries of Fuxian Lake. J Yuxi Teach Coll. 2006;22:64–68.

[10] Schindler DW. Carbon, Nitrogen, and Phosphorus and the Eutrophication of Freshwater Lakes1. J Phycol. 1971;7:321–329. 10.1111/j.1529-8817.1971.tb01527.x

[11] Schindler DW, Hecky RE, Findlay DL, Stainton MP, Parker BR, Paterson MJ, et al Eutrophication of lakes cannot be controlled by reducing nitrogen input: Results of a 37-year whole-ecosystem experiment. Proc Natl Acad Sci. 2008;105:11254–11258. 10.1073/pnas.0805108105 18667696 PMC2491484

[12] Ryther JH, Dunstan WM. Nitrogen, Phosphorus, and Eutrophication in the Coastal Marine Environment. Science. 1971;171:1008–1013. 10.1126/science.171.3975.1008 4993386

[13] Keatley BE, Bennett EM, MacDonald GK, Taranu ZE, Gregory-Eaves I. Land-Use Legacies Are Important Determinants of Lake Eutrophication in the Anthropocene. Finkel Z, editor. PLoS ONE. 2011;6:e15913 10.1371/journal.pone.0015913 21264341 PMC3018476

[14] Yuxi Federation of social science circles. Research Report on the Development of Yuxi. Kunming:China: Yunnan People’s Press; 2013.

[15] Bu H, Tan X, Li S, Zhang Q. Temporal and spatial variations of water quality in the Jinshui River of the South Qinling Mts., China. Ecotoxicol Environ Saf. 2010;73:907–913. 10.1016/j.ecoenv.2009.11.007 20047760

[16] Chang N-B. Sustainable water resources management under uncertainty. Stoch Environ Res Risk Assess. 2005;19:97–98. 10.1007/s00477-004-0217-1

[17] Kolovos A, Christakos G, Serre ML, Miller CT. Computational Bayesian maximum entropy solution of a stochastic advection-reaction equation in the light of site-specific information: BME SOLUTION TO ADVECTION-REACTION. Water Resour Res. 2002;38:54–1–54–17. 10.1029/2001WR000743

[18] Huang F, Wang X, Lou L, Zhou Z, Wu J. Spatial variation and source apportionment of water pollution in Qiantang River (China) using statistical techniques. Water Res. 2010;44:1562–1572. 10.1016/j.watres.2009.11.003 19944441

[19] Li S, Xia X, Tan X, Zhang Q. Effects of Catchment and Riparian Landscape Setting on Water Chemistry and Seasonal Evolution of Water Quality in the Upper Han River Basin, China. Mozumdar S, editor. PLoS ONE. 2013;8:e53163 10.1371/journal.pone.0053163 23349700 PMC3551924

[20] Wang Q, Wu X, Zhu C, Yang H. Temporal and Spatial Distribution Characteristics of Nitrogen on the West Bank of the Fuxian Lake. J Yuxi Norm Univ. 2013;29:9–12.

[21] Liu X, Yu X, Huang Y, Zhang L. Evaluation on Ecological Security Variation Trend in Weinan City from 1985 to 2003 by Spearman Rank Related Coeffident Method. J Anhui Agric Sci. 2010;38:16341–16342.

[22] Li X, Wu X, Huang L, Qin Z. Analysis and Evaluation on Water Quality Present Situation and Change Tendency of Fisheries Area of Lianzhou Bay, Hepu County, Guangxi Based on the Rank correlation Coefficient. J Anhui Agric Sci. 2011;39:18112–18113,18169.

[23] Wan L, Mao B. Batch Calculation of Spearman Rank Correlation Coefficient. Environ Prot Sci. 2008;34:53–55.

[24] Li X, Wu X, Qin Z, Yang S. Evaluation based on the rank correlation coefficient for plant plankton biomass change tendency of fish reproduction spot in Dongta. Guangdong Agric Sci. 2011;121–122.

[25] Wang Q, Li Y, He J, Xu X, Wu X. Analysis of water quality variation trend in lake inlet river on north of Lake Dianchi. Environ Sci Technol. 2012;35:191–194.

[26] Gao W, Chen Y, Xu M, Guo H, Xie Y. Trend and driving factors of water quality change in Lake Fuxian (1980–2011). J Lake Sci. 2013;25:635–642.

[27] Ministry of Environmental Protection of the People’s Republic of China. Technical Guideline for Surface Water Environmental Quality Assessment. Beijing: China: Environmental Science Press; 2008.

[28] Kowalkowski T, Zbytniewski R, Szpejna J, Buszewski B. Application of chemometrics in river water classification. Water Res. 2006;40:744–752. 10.1016/j.watres.2005.11.042 16442142

[29] Xu HS, Xu ZX, Wu W, Tang FF. Assessment and Spatiotemporal Variation Analysis of Water Quality in the Zhangweinan River Basin, China. Procedia Environ Sci. 2012;13:1641–1652. 10.1016/j.proenv.2012.01.157

[30] S. Shrestha FK. Assessment of surface water quality using multivariate statistical techniques: A case study of the Fuji river basin, Japan. Environ Model Amp Softw. 2007;464–475. doi:10.1016/j.envsoft.2006.02.001

[31] Wang Y, Wang P, Bai Y, Tian Z, Li J, Shao X, et al Assessment of surface water quality via multivariate statistical techniques: A case study of the Songhua River Harbin region, China. J Hydro-Environ Res. 2013;7:30–40. 10.1016/j.jher.2012.10.003

[32] Wang X, Cai Q, Ye L, Qu X. Evaluation of spatial and temporal variation in stream water quality by multivariate statistical techniques: A case study of the Xiangxi River basin, China. Quat Int. 2012;282:137–144. 10.1016/j.quaint.2012.05.015

[33] Singh EJK, Gupta A, Singh NR. Groundwater quality in Imphal West district, Manipur, India, with multivariate statistical analysis of data. Environ Sci Pollut Res. 2013;20:2421–2434. 10.1007/s11356-012-1127-2 22935861

[34] Parelho C, Rodrigues AS, Cruz JV, Garcia P. Linking trace metals and agricultural land use in volcanic soils—A multivariate approach. Sci Total Environ. 2014;496:241–247. 10.1016/j.scitotenv.2014.07.053 25093299

[35] Su S, Zhi J, Lou L, Huang F, Chen X, Wu J. Spatio-temporal patterns and source apportionment of pollution in Qiantang River (China) using neural-based modeling and multivariate statistical techniques. Phys Chem Earth Parts ABC. 2011;36:379–386. 10.1016/j.pce.2010.03.021

[36] Singh KP, Malik A, Sinha S. Water quality assessment and apportionment of pollution sources of Gomti river (India) using multivariate statistical techniques—a case study. Anal Chim Acta. 2005;538:355–374. 10.1016/j.aca.2005.02.006

[37] The tobacco industry Office of Yuxi Municipal People’s Government. Yuxi tobacco soil management and fertilization Kunming:China: Yunnan Science and Technology Press; 2008.

[38] Nachtergaele Freddy, van Velthuizen Harrij, Verelst Luc. Harmonized World Soil Database. Food and Agriculture Organization of the United Nations; 2008.

[39] State Environment Protection Bureau Of China. Water And Wastewater Analysis Method. Beijing: China: Environmental Science Press; 2002.

[40] Bergström L, Brink N. Effects of differentiated applications of fertilizer N on leaching losses and distribution of inorganic N in the soil. Plant Soil. 1986;93:333–345. 10.1007/BF02374284

[41] Statistical Bureau of Yunnan Province. Yunnan statistical yearbook 2013 Beijing: China: China statistics press; 2013.

[42] Brookes PC, Heckrath G, de Smet, Hofman G, Vanderdeelen J. Losses of phosphorus in drainage water CAB INTERNATIONAL; 1997 pp. 253–271.

